# Accessory Toxins of *Vibrio* Pathogens and Their Role in Epithelial Disruption During Infection

**DOI:** 10.3389/fmicb.2018.02248

**Published:** 2018-09-20

**Authors:** Diliana Pérez-Reytor, Victor Jaña, Leonardo Pavez, Paola Navarrete, Katherine García

**Affiliations:** ^1^Instituto de Ciencias Biomédicas, Facultad de Ciencias de la Salud, Universidad Autónoma de Chile, Santiago, Chile; ^2^Facultad de Medicina Veterinaria y Agronomía, Universidad de Las Américas, Santiago, Chile; ^3^Departamento de Ciencias Químicas y Biológicas, Universidad Bernardo O’Higgins, Santiago, Chile; ^4^Laboratorio de Microbiología y Probióticos, Instituto de Nutrición y Tecnología de los Alimentos, Universidad de Chile, Santiago, Chile

**Keywords:** Zot, RTX, Ace, toxins, tight junctions, intestinal epithelia, *Vibrio*

## Abstract

Gastrointestinal episodes associated with *Vibrio* species have been rising worldwide in the last few years. Consequently, it is important to comprehend how occurs the production of diarrhea, to establish new preventive and therapeutic measures. Besides the classical CT and TCP toxins, Zot, RTX, and Ace among others have been deeply studied in *V. cholerae.* However, in other *Vibrio* species of clinical interest, where some of these toxins have been reported, there is practically no information. Zot activates a cascade of signals inside of the cell that increase the permeability of epithelial barrier, while RTX causes depolymerization of the actin cytoskeleton and Ace increases the permeability of intestinal cell monolayers. The goal of this study is to acquire information about the distribution of these toxins in human pathogenic *Vibrios* and to review the progress in the study of their role in the intestinal epithelium during infection.

## Introduction

Gastrointestinal pathogens invade or disrupt the intestinal barrier by the action of secreted toxins. They can alter cell physiology by multiple mechanisms, being directly responsible for the pathology of the disease or favoring other processes such as manipulation of the host immune response, escape from the intracellular environment and penetration of host barriers, among others (Ugalde-Silva et al., 2016). The *Vibrionaceae* family includes several species of major importance in the clinical field that are able to cause gastroenteritis. Among these, *Vibrio cholerae* is the classical pathogen carrying an arsenal of diverse toxins that produce illness. This bacterium is the agent responsible for cholera, an infection of the small intestine whose classical symptoms are a watery diarrhea, vomiting and dehydration; it is associated with million cases and several deaths around the world each year. (Vezzulli et al., 2016; Plaza et al., 2018). Serotypes O1 and O139, mainly responsible for acute diarrheal disease, possess two main virulence genes, the cholera toxin (CT) and the toxin-coregulated pilus (TCP). However, other strains (non-O1/non-O139) that cause sporadic cases of diarrhea have toxigenic potential attributed to secretion systems (T3SS and T6SS) and other accessory toxins like zonula occludens toxin (Zot) (Chatterjee et al., 2009). Additionally, they have other genes, coding to hemolysins and repeats in toxin (MARTX) which have role helping to the colonization of the intestine (König et al., 2016). Additionally, accessory cholera enterotoxin (Ace) causes fluid secretion in ligated rabbit ileal loops (Trucksis et al., 1993) while cholix toxin (ChxA) is an exotoxin which has been characterized as a member of the eukaryotic elongation factor 2-specific ADP-ribosyltransferase toxins (König et al., 2016). *V. cholerae* is not the only *Vibrio* producing toxins; other members of the *Vibrionaceae* family also produce toxins that generate illness. Although it is not its primary pathology, *V vulnificus* is capable of producing gastroenteritis among its clinical manifestations. This pathogen secretes a number of exotoxins and enzymes (Liu et al., 2007), among which the cytolysin VvhA (Kim et al., 2017) and metalloprotease Vvp (Lee et al., 2008; Chen et al., 2017) are the most important join to RTX. This last toxin has been strongly associated with the virulence of this organism (Lee et al., 2007, 2008). It is suggested that this toxin might protect the bacterium from phagocytosis, promoting their survival. Surprisingly, sequencing of *V. vulnificus* MO6-24/O strain isolated from a septicemic patient showed that this strain possessed a phage-related gene cluster containing *ace* and *zot* genes (Park et al., 2011). Similarly, a recent study showed that some strains of *Vibrio parahaemolyticus* lacking the classical toxins TDH and TRH possess genomic islands and prophage elements containing RTX, Zot and Ace toxins. Zot has also been found in prophage f237 of the pandemic strain and in prophage-like elements in *V. coralliilyticus* and *V. anguillarum*, suggesting that the horizontal gene transfer (HGT) associated to phages coding *zot* occurs frequently among *Vibrio* species (Castillo et al., 2018). If pathogenicity genes are combined by HGT at high frequency the probability of generate new virulent species increases (Nishibuchi and Kaper, 1995), which is favored in estuaries and marine environments which represent a extensive pool of virulence genes associated to species of the genus *Vibrio* (Ceccarelli et al., 2013; Khouadja et al., 2014).

Martine Urtaza and their collaborators recently shown that the risk of infections associated to *Vibrio* species is rising in many parts of the world (Martinez-Urtaza et al., 2010), and as a consequence of global warming, it is expected that cases would be increasing in frequency and intensity. The broad spectrum of toxins found in the genomes of *Vibrios* draws attention to which toxins are essential in the development of gastroenteritis and which others are responsible for extra-intestinal infections. An unequivocal knowledge of the mechanisms responsible of the development of infection is crucial to establish new precautionary measures and treatments. The goal of this study is to gain understanding about the distribution of accessory toxins in human pathogenic *Vibrios* and to review the advancement in the study of the role of these toxins in the intestinal epithelium during infection by different *Vibrio* pathogens.

## The Intestinal Barrier: The Role of Tight Junctions (TJs) Proteins

**FIGURE 1:**
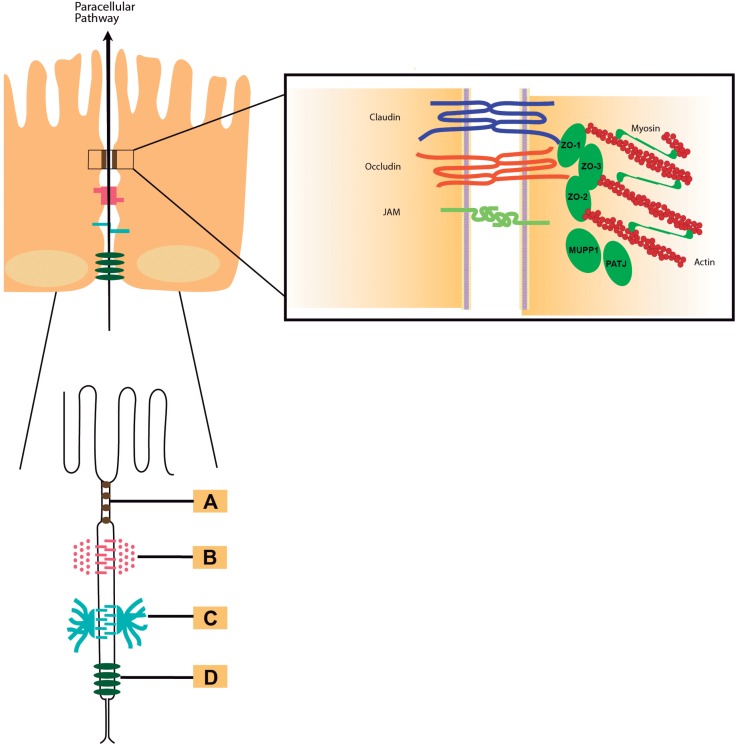
Intercellular junctions. Schematic draw of the intercellular junctions showing **(A)** tight junction, **(B)** adherens junction, **(C)** desmosome, and **(D)** gap junction. *In detail to the right of the figure*: Tight junctions are multiprotein complexes containing members of the claudins (in blue), occludin (in red), and junctional adhesion molecules (JAMs, in green color) families.

The intestines are organs in the digestive system tract involved in the uptake of nutrients and water (Farhadi et al., 2003). They also represent a barrier against pathogens of the outside environment. Due to the protection function, intestinal permeability is a highly regulated dynamic process. The intestinal barrier is mainly composed of three layers: mucus, epithelia, and the lamina propria (König et al., 2016). The epithelial layer is a single layer of cells, which selectively regulate the absorption of nutrients and prevent or modulate the access of microorganisms, toxins and other macromolecules from the intestinal lumen (Salvo-Romero et al., 2015). The epithelial cells that make up the barrier are maintained together by specialized intercellular junctions: desmosomes, adherens junctions (AJs) and tight junctions (TJs) (Citi et al., 2014; Ugalde-Silva et al., 2016). All of them, in addition to the intestinal microbiota and immunogenic mechanisms, possess a joint crucial role to maintain the appropriate function of the intestinal barrier. Consequently, the well-functioning of this barrier strongly depends on the normal function of the paracellular pathway (Farhadi et al., 2003). Tight junctions, also called *zonulae occludentes*, are the intercellular junctions most apically located (**Figure 1**; Gopalakrishnan et al., 2009) and interruption of these junctions contributes to the inflammatory response because of increased antigenic penetration (Al-Sadi et al., 2013). These junctions also regulate the selective paracellular permeability to solutes, ions, water and various macromolecule (Van Itallie and Anderson, 2014) and the entry of microorganisms inhabiting in the intestinal mucosa (Ugalde-Silva et al., 2016). This barrier function is conferred by a large and diverse group of transmembrane proteins mainly composed of proteins claudins, occludins, and the zonula occludens-associated (ZO) proteins 1, 2, and 3 (Lee et al., 2003) among others. The cytosolic scaffold protein ZO-1 directly and indirectly couples occludin and claudins to the other cytoplasmic TJ proteins (Turner et al., 2014) and the actin cytoskeleton (**Figure 1**).

TJ assembly and disassembly is a dynamic process that involves endocytosis, migration and recycling in all epithelia. The regulation of TJs occurs by PKC activation affecting stability in the dynamic TJ complex. This is coincident with increases in paracellular permeability mediated by TJ (Turner et al., 2014). Several pro-inflammatory cytokines can also modulate TJ dynamics causing disruption of the intestinal TJ barrier and resulting in an increase of intestinal TJ permeability (Al-Sadi et al., 2009, 2013, 2016) while anti-inflammatory cytokines were shown to promote intestinal TJ barrier function (Al-Sadi et al., 2016). Other factors that impact TJ dynamics and assembly includes intracellular cAMP and calcium imbalance among others, which act through their varied effects on cellular kinases (König et al., 2016). Another structure indispensable for the integrity of paracellular pathway is the cytoskeleton. The cytoskeleton is the structure that maintains the shape and internal organization of the cells, besides giving mechanical support to carry on cellular movement and division. The interaction of TJ proteins with the actin maintain the structure of cytoskeletal, which is essential for the proper regulation of barrier functioning (González-Mariscal et al., 2013).

## The Interaction Between the Intestinal Barrier and Pathogenic Microorganisms

The epithelia comprise structures adapted to protect the tissues from pathogenic microorganisms, antigens and/or proinflammatory factors (Farhadi et al., 2003; Sousa et al., 2005). Conversely, pathogens have generated diverse strategies that disrupt the components that maintain the structure of epithelia and spread to various tissues (Sousa et al., 2005; Dubreuil, 2017). Pathogens can secrete enzymes that affect the extracellular part of junction components or toxins acting within the cell, disrupting intercellular junctions. Other can inject effector proteins into the host cell cytoplasm, altering cell functions by acting on cell signaling pathways (Ugalde-Silva et al., 2016). Invasive pathogens can destabilize the junctions by inducing a signaling cascade that lead to proinflammatory response or directly targeting the intercellular junction. The cytoskeleton is also a target for toxins by direct and indirect modifications through covalent or non-covalent mechanisms, respectively (Barbieri et al., 2002). The mechanism of action is different among toxins, some of them shift the equilibrium between F- (polymerized) and G-actin (monomeric) (Kudryashov et al., 2008), while other can affect directly actin.

The interactions between the enteric pathogenic microorganisms and their hosts is of great interest to try to understand several mechanisms of infectious diseases. The relationship between the toxic products of bacteria and diarrheal diseases has been studied extensively (Guttman and Finlay, 2009). Although one of the function of the epithelial barriers is to block the access of many organisms, certain pathogens have evolved to alter this barrier. In this context, most of gastrointestinal pathogens lead to intestinal secretion by elaboration of toxins or invasion (Fasano, 2012). They can use tight junction proteins as receptors for their internalization or destroy the junctions to enter to the underlying tissue. As a result, the altering of tight junctions elicit inflammatory cascades causing diarrhea as the ultimate goal (Dubreuil, 2017).

## The *V. cholerae* Pathogenesis: The Cholera Toxin

Since cholera is a global disease responsible for several cases of diarrhea and deaths around the world, the mechanisms of pathogenicity of this pathogen have been deeply studied (WHO (2015) *Cholera: Fact Sheet No. 107).* The cholera disease is an acute infectious diarrhea whose transmission occurs mainly through contaminated water or foods. Once this pathogen interacts with the epithelial cells of the human small intestine a massive watery efflux occurs characteristic of cholera diarrhea, which functions to disperse *V. cholerae* back into the environment. Therefore, the diarrhea causes severe dehydration and in many cases the death of infected people (Cordero et al., 2006).

The pathogenesis of cholera is a multifactorial process involving several genes that encode virulence factors that help the bacteria in colonization and the expression of the cholera toxin (CT). Each CT molecule is composed of one A subunit plus five B subunits. The B subunits bind to the ganglioside GM1 receptors in the epithelial cells of the intestinal mucosa. After binding, subunits A1 and A2 are separated, which facilitates the entry of component A1 into the cell. Component A1 of the CT stimulates the production of the enzyme adenyl cyclase, involved in the production of cyclic adenosine monophosphate (cAMP) (Bharati and Ganguly, 2011). The high intracellular concentrations of cAMP alter the transport of electrolytes through the cell membrane, activating the cystic fibrosis transmembrane conductance regulator (CFTR) and resulting in secretion of chloride ions into the lumen. The receptor for the CT is composed of flexible homopolymers of the monomeric form of the toxin-coregulated pili (TCP) pilin subunit TcpA that self-associates, holding cells together in microcolonies. All the *ctxAB* operon is part of the genome of the filamentous bacteriophage CTXf, lysogenized in the bacterium (Fasano et al., 1991; Waldor and Mekalanos, 1996; Olivier et al., 2007). Besides CT, it also carries the genes involved in the morphogenesis of the bacteriophage (*psh, cep, orfU*, and *ace*) and a gene that encodes a protein necessary for the assembly of the virion (*zot*). Both gene products, Zot and Ace, are also able to contribute to *V. cholerae* pathogenesis by inducing changes in the intestinal barrier (Fasano et al., 1991; Baudry et al., 1992; Fasano, 2012; Chatterjee et al., 2015)

### The Zot Toxin

**FIGURE 2:**
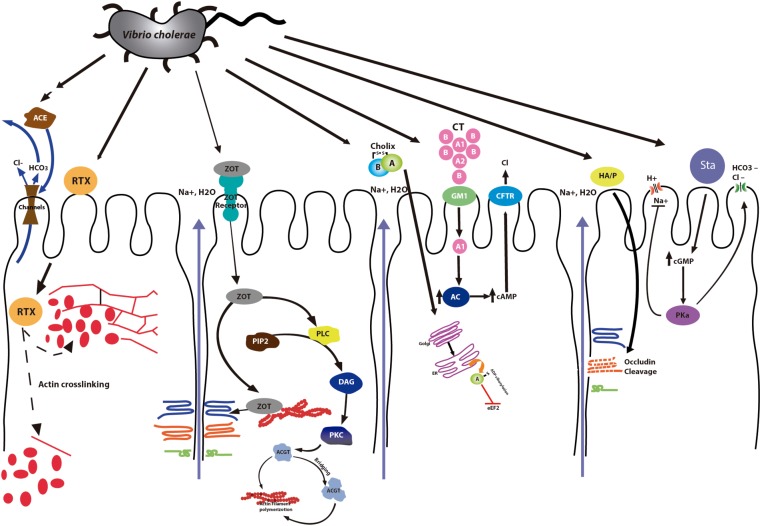
*V. cholerae* infection. Mechanism of action of *V. cholerae* toxins. The Cholera Toxin (CT) binds to the receptor (Ganglioside GM1) and enhances adenylate cyclase (Ac) activity, increasing cAMP. Elevated intracellular concentrations of cAMP activate the cystic fibrosis transmembrane conductance regulator (CFTR), resulting in secretion of chloride ions into the lumen. Zonula occludens toxin (Zot) affects the structure of the TJs increasing the permeability. Accesory cholera enterotoxin (ACE) stimulates Ca^2+^-dependent Cl^-^/HCO3^-^ secretion in intestinal cells. Heat stable enterotoxin (Sta) leads to the increase of cGMP inhibiting the regulatory mechanism of Na^+^/Cl^-^. The Repeats in toxin (RTX) leads to the depolymerization of stress fibers. Cholix Toxin (ChT) inhibits protein synthesis. Finally, although Hemagglutinin (HA)/protease (HA/P) is not a toxin, it produces the cleavage of occludin into two distinct fragments, affecting the paracellular pathway of intestinal epithelial cells in culture. The accessory toxins Zot, Ace and RTX are also found in the genome of other *Vibrio* species.

Zonula occludens toxin (Zot) was discovered in *V. cholerae* when live oral vaccines, constructed by deletion of *V. cholerae* sequences encoding the A subunit of the CT, were applied to volunteers. These strains still provoked diarrhea (mild to moderate) in some volunteers due to the presence of another toxin that interacts with tight junctions affecting the paracellular pathway (Fasano et al., 1991). Zot is also encoded in the bacteriophage CTXf; its N-terminal side is involved in phage morphogenesis. In fact, a *zot* mutation impairs the release of phage particles into the culture supernatant (Uzzau et al., 1999). The C-terminal is secreted into the intestinal lumen after clivage (Fasano et al., 1991; Schmidt et al., 2007). Studies have shown that a smaller fragment of 12 kD is the fragment of Zot with biological activity (Di Pierro et al., 2001). The enterotoxic and permeabilizing effect of Zot on rabbit small intestine was first shown by Fasano and collaborators. They described that Zot has a regional effect which varies in the different segments of rabbit intestine (Fasano et al., 1997). Later, Uzzau and coworkers demonstrated that Zot induces a transitory reduction in transepithelial electrical resistance and an increase in transepithelial flux, increasing the permeability of TJs (Uzzau et al., 2001). They also identified the Zot region required for receptor binding. The Zot receptor is a protein that is located on the cell surface and acts by modulating the cytoskeleton and the tight junction complex inside of the cell (Uzzau et al., 2001). Currently it is known that Zot is positioned in the cell envelope of bacteria (Di Pierro et al., 2001; Salama et al., 2004) and that its action is mediated by intracellular signaling that leads to a reduces the actin filaments (changing the F- and G-actin pools, **Figure 2**). The change of actin microfilaments increases intestinal epithelial permeability by affecting the TJs (Lee et al., 2003; Goldblum et al., 2011). It has been demonstrated that Zot increases the transport of diverse macromolecules such as insulin, sucrose and acyclovir across several surfaces, including blood-brain barrier and mucosal (Gopalakrishnan et al., 2009).

### The ACE Toxin

The “virulence cassette” of *V. cholera*e includes genes encoding CT and Zot but also a third toxin called Ace (Trucksis et al., 1993; Anvari et al., 2012). This last is an integral membrane protein consisting of 96 amino acids which alters ion transport, causes accumulation in ligated rabbit ileal loops and is responsible for mild diarrhea (Chatterjee et al., 2011). Not much is known about this toxin because of the low amount produced by *V. cholerae*. Ace stimulates Ca^2+^ -dependent Cl^-^ /HCO^3-^ symporters in a colonic carcinoma T84 monolayer cell model, creating a potential difference across the membrane (Chatterjee et al., 2011, 2015).

### Heat Stable Enterotoxin

Heat stable enterotoxin (ST) is a peptide composed of 17 aminoacids that induces Ca^2+^ release from the cell in response to IP3, leading to the activation of guanylyl cyclase and the production of cGMP (Al-Majali et al., 2007). The increase in intracellular cGMP inhibit the regulatory mechanism of Na ^+^/Cl^-^ eliciting secretory diarrhea (Al-Majali et al., 2000).

### Repeats in Toxin

The repeats-in-toxin (RTX) proteins of Gram-negative bacteria have in common the mode of export via the Type I-Secretion System and classical C-terminal GD-rich repeats (Linhartová et al., 2010). The RTX family generally consists of hemolysins and cytolysins with molecular masses ranging between 40 and more than 600 kDa, that display a variety of activities (Linhartová et al., 2010; Dolores et al., 2015) and are activated by acylation post-translationally (Frey and Kuhnert, 2002). RTX pore-forming toxins involved in bacterial pathogenesis, characterized by repeats of a glycine and aspartate-rich, calcium-binding sequence motif are the members of RTX family most studied and known (Pei and Grishin, 2009; Satchell, 2011). The RTX toxins are four genes of two operons: *rtxA* encoding the toxin; *rtxB/rtxE*, an ATP-binding cassette transporter of RtxA; *rtxC*, an acylase of RtxA; and *rtxD*, with no clear function yet (Linhartová et al., 2010). They can act in a synergistic way, causing damage and triggering the liberation of inflammatory molecules (Boardman and Fullner Satchell, 2004). *In vitro*, these toxins show hemolytic and cytotoxic activity which induce damage to the membrane, osmotic changes and finally, cell death by lysis (Wiles and Mulvey, 2013). However, in host cells the cytotoxicity of RTX toxins produces also apoptosis, although the mechanisms is not understood (Wiles and Mulvey, 2013). The best characterized are the multifunctional-autoprocessing RTX (MARTX) toxins, a subgroup of very large RTX proteins (range from 3,500 to 5,300 aminoacid residues) with multiple activities and which constitute a combination of secreted toxins and multi-effector delivery systems (Woida and Satchell, 2018). These proteins are encoded by *V. cholerae* (VcRtxA), *V. vulnificus* and other pathogens (Lee et al., 2008). In *V. cholerae* the MARTX_V c_ (MARTX of *V. cholerae)* is found in practically all strains (including environmental and clinical isolates and pandemic strains) (Menestrina et al., 1994; Chatterjee et al., 2008). This toxin acts by avoiding the elimination of *V. cholerae* from the intestine at the beginning of infection (Satchell, 2015). In this way, *V. cholerae* MARTX_V c_, contributes to the pathogenesis of cholera in model systems (Kudryashov et al., 2008) although not possess the cytolytic (or hemolytic) activities displayed by other RTX toxins. MARTX_V c_, like CT and pore-forming toxin hemolysin, is associated with the ability to establish a persistent intestinal infection by bacteria (Prochazkova and Satchell, 2008; Olivier et al., 2009). It has been shown that MARTX_V c_ directly catalyzes a covalent cross-linking of G-actin (monomer) into oligomeric chains, causing cell rounding by disassembly of the actine stress fibers in culture (Sheahan et al., 2004; Cordero et al., 2006). Two distinct virulence activity domains in MARTX_V c_ are responsible for the toxin effect. Actin cross-linking domain causes irreversible disassembly of the cytoskeleton by directly catalyzing the covalent cross-linking of monomeric G-actin. The Rho GTPase inactivation domain causes inactivation of small GTP-bound Rho, Rac, and Cdc42, resulting in depolymerization of actin (Sheahan et al., 2004; Kudryashov et al., 2008; Prochazkova and Satchell, 2008). In 2017, Chen and coworkers showed that MARTXs contain repeated motifs. In the C-terminus there are GD-rich repeats whereas the repeats at the N-terminus are required for toxin secretion and effector translocation (Chen et al., 2017).

### Cholix

Cholix toxin (Cholix, ChxA) is a newly identified virulence factor reported in non-pandemic strains (non O1/non O139 *V. cholerae* strains; Purdy et al., 2010). Cholix is a 70 kDa ADP-ribosyltransferase toxin that translocates into cells by receptor-mediated endocytosis (Ogura et al., 2011; Ogura et al., 2017) and utilizes eukaryotic elongation factor 2 (eEF2) as a substrate (Ogura et al., 2017). The transference of an ADP-ribose group from NAD ^+^ to a diphthamide in eEF2 mediated by cholix inhibit the synthesis of protein producing finally cell death (Jørgensen et al., 2008). Because of the similarity with exotoxin A, it is suggested that cholix toxin enters eukaryotic cells by endocytosis where it is cleaved and the catalytic domain is released to the cytosol where it exerts its effect. This toxin is active against mammal and crustacean cells (Fernandez and Alonso, 2009), suggesting that it plays a role in the survival in their natural environment. Interestingly, the study of Awasthi et al. (2013) showed that there are three types of cholix and none of them caused enterotoxicity in rabbits, however, two of them caused extensive damage in internal organs in mice, suggesting that cholix is associated with extraintestinal infections at least in one animal model (Awasthi et al., 2013)

### Hemagglutinin/Protease (HA/P)

Although HA/P, the *V. cholerae* hemagglutinin/protease, is not a toxin, it has been described that can play a role during colonization of the intestine (Lutfullah et al., 2008; Shinoda and Miyoshi, 2011). HA/P is Zn-dependent metalloprotease with mucinase activity, encoded by *hapA* (Silva et al., 2006; Lutfullah et al., 2008). It exhibit several activities including modification of toxins, degradation of the mucus barrier and acting on TJ-associated proteins, Cleavage of occludin by HA/P resulted in rearrangement of ZO-1, the F-actin cytoskeleton and disruption of paracellular barrier function (Silva et al., 2006; Benitez and Silva, 2016). HA/protease also shows homology to *V. vulnificus* elastase (VvpE) at aminoacidic level, which is important due to VvpE contributes to local tissue damage during infections produced by this *Vibrio* (Lee et al., 2015).

## Variation of RTX and CT and Their Impact in the *V. cholerae* Pathogenicity

The first global spread of cholera disease, occurred from the early 1960s through the middle 1990s, was mainly associated to the El Tor strains of *V. cholerae*. However, since the late 1990s, a lineage known as the altered El Tor (AET) *V. cholerae* has come to predominate as the major cause of human cholera disease (Satchell et al., 2016) and it has been associated to severe cases of diarrhea. Interestingly, although *V. cholerae* possess diverse variants of MARTX toxin associated to environmental strains able to produce disease, the AET *V. cholerae* strains have an inactivated MARTX toxin gene. This inactivation is explained because the *rtxA* toxin gene possesses one SNP that introduces a stop codon, resulting in a truncated protein (Dolores and Satchell, 2013). Despite the absence of RTX, these strains are more virulent (Satchell et al., 2016), questioning if RTX is necessary for the pathogenicity of Vibrio. Some authors have proposed that this very large toxin is eliminated once it is not necessary, because it may be detrimental to growth due to energy costs (Dolores and Satchell, 2013). Besides, RTX is fully redundant in function with a pore-forming hemolysin (Olivier et al., 2009).

Interestingly, the null mutant of *rtxA* was the genetic background for following emergence of the *ctxB7* allele, a point mutation in *ctxB* that created a CtxB with Asparagine at aminoacid 20 (H20 to N20 change) (Dolores and Satchell, 2013). The functional consequence of this change is unknown, but it is suggested that it may affect the maturation of the toxin (Satchell et al., 2016). This is significant considering that the severity of cholera is associated with the production of CT, as El Tor strains carrying ctxB-1 were associated with more severe symptoms (Siddique et al., 2010). Additional changes in the CTX gene resulted later in substitution of *ctxB*1 with *ctxB*7 (Rashid et al., 2016).

Strains carrying *ctxB* allela were first detected in Odisha, India, in 2007 (Kumar et al., 2009) and have been transmitted globally, reaching Cameroon (Africa) in 2009, Nepal in 2010 and Haiti in 2010 (Quilici et al., 2010; Hendriksen et al., 2011) where they produced a devastating outbreak.

## Distribution of Toxins in Other Non-Cholerae *Vibrio*

Diarrhea associated with seafood consumption are mainly associated to pathogenic *V. parahaemolyticus* strains (García et al., 2009; Letchumanan et al., 2017). Several characteristic virulence associated factors of this pathogen such as hemolysins TDH (thermostable direct hemolysin), TRH (TDH-related hemolysin) and the secretion systems (Broberg et al., 2011; Ceccarelli et al., 2013; Raghunath, 2014) have been deeply studied. Nonetheless, the pathogenesis of *V. parahaemolyticus* is still not fully understood. Some studies have reported that environmental isolates of *V. parahaemolyticus* lacking most characteristic virulence factors (*tdh*, *trh*, and T3SS-2) are able to produce cellular damage (Mahoney et al., 2010; Castillo et al., 2018; Wagley et al., 2018) while others have reported clinical strains with absence of all principal virulence factors isolated with patients with gastroenteritis (García et al., 2013). These results indicate that classic virulence factors are not sufficient to explain the cytotoxicity and enterotoxicity of pathogenic *V. parahaemolyticus* strains and suggest that a novel virulence factor (or more than one) could be responsible for pathogenicity. A comparative genomic analysis of environmental and clinical strains of *V. parahaemolyticus* revealed the absence of most of the classical toxins and virulence factors described for *V. parahaemolyticus* in cytotoxic strains, but instead they had novel and uncharacterized toxins in the accessory genome, mainly associated with prophages and pathogenicity islands. Bioinformatics analysis revealed the presence of prophage-like elements which encoded a putative Zot-like enterotoxin (Castillo et al., 2018). Interestingly, three phages contained three different *zot* sequences, suggesting high diversity within the same species. However, although *V. parahaemolyticus* and *V. cholerae* Zot shared only 24% amino acid identity, they share some conserved regions (Castillo et al., 2018), suggesting that the structure acquired by Zot is more important than the sequence. Additionally, other *V. parahaemolyticus* strain, PMA 1.15, contained a prophage carrying a putative RTX toxin in addition a novel genomic island containing DNase and RTX toxin genes (see Figure 4 in Castillo et al., 2018). However, until this manuscript appeared, no studies about the function of Zot or RTX in *V. parahaemolyticus* were published.

*V. vulnificus*, another important human pathogen, is distributed worldwide in estuaries and marine environments, where is associated to food-borne and wound infections exhibiting high mortality (Ziolo et al., 2014; Kim et al., 2017), which exceeds 50%, and can increases to more than 90% in patients in serious condition (shock) (Horng-Ren et al., 2011; Chen et al., 2017). This bacterium produces several virulence factors that cause disease, including cytolysin VvhA, metalloprotease Vvp, flagella and RtxA toxin among others (Lee et al., 2008; Chen et al., 2017). RtxA1 toxin is the most potent cytotoxic virulence factor (Lee et al., 2007) of *V. vulnificus* (Gavin et al., 2017). It has been shown that it exerts dramatic effects on cytoskeletal rearrangement, contact cytotoxicity, hemolysis (inducing the apoptotic death of human epithelial cells (Kim et al., 2017)), invasion and lethality to mice, showing that it is a multifunctional virulence factor of *V. vulnificus* (Lee et al., 2007). Like that of *V. cholerae*, the RTX of *V. vulnificus* has multiple domains and it is autoprocessed (Jeong and Satchell, 2012; Gavin and Satchell, 2015; Chen et al., 2017; Kim et al., 2017). Mutants on this toxin are significantly attenuated for virulence (Liu et al., 2007; Kim et al., 2017). A *V. vulnificus* null mutant in the *rtxA* gene constructed by Lee et al. (2007) exhibited decreased cytotoxic activity, using NT-407 intestinal epithelial cells as model. MARTX_V v_ (multifunctional-autoprocessing repeats-in-toxins toxin of *V. vulnificus)* and the cytolysin VvhA play a role in growth of bacteria *in vivo*, therefore the presence of both factors is directly correlated with mouse mortality (Jeong and Satchell, 2012). The importance of N-termini and C-termini of MARTX_V v_ has been shown by Kim et al. (2015). A deletion in the C-terminal region blocked toxin secretion from the bacterium and consequently a reduction in the cytotoxicity of bacteria. In contrast, a deletion in the N-terminal domain completely abolished necrosis (Kim et al., 2015).

Interestingly, the complete genome sequence of *V. vulnificus* MO6-24/O isolated from a septicemic patient showed that this particular strain contains 272 specific genes, including phage-related genes. The gene cluster of the bacteriophage contains *ace* and *zot*, revealing genetic diversity resulting from extensive gene transfer (Park et al., 2011).

Finally, although it is not considered as an important human pathogen until today, recent observations have indicated that *V. mimicus* may cause epidemic diarrhea. Some of these strains contains the “cholera virulence cassette” containing genes encoding Zot, Ace and a core encoded pilus as well as CT (Shi et al., 1998, 2000).

## Conclusion

Risk of gastrointestinal infections associated with *Vibrio* species has been rising worldwide as a consequence of global warming. However, to date the mechanisms involved in the production of diarrhea by some *Vibrio* species is not completely understood. *V. cholerae* has an arsenal of toxins, the most important being the classical CT, but also Zot, RTX and Ace contribute to the enterotoxicity of this pathogen, while cholix produces extraintestinal effects (**Table 1**). In *Vibrio* the HGT plays a significant role in the transmission of genes. In fact, bacteriophages and other mobile elements containing Zot, Ace and also RTX have been reported in the genomes of clinical and environmental strains of *V. parahaemolyticus* that exhibited high cytotoxicity. Also, a bacteriophage containing *zot* and *ace* genes was reported in a strain of *V. vulnificus* isolated from a patient with septicemia. Similarly, some strains of *V. mimicus* possess the virulence cassette of *V. cholerae.* Regrettably no studies of the characterization and function of these toxins were found until this manuscript was finished, and we do not know if they contribute to the pathogenicity of *V. parahaemolyticus* and *V. vulnificus*, and if they do, in what way. In the light of the facts, it seems urgent to know the molecular mechanisms behind the mode of action of these novel enterotoxins in *Vibrios* other than *V. cholerae*.

**Table 1:** Classical and accessory toxins of *Vibrio cholerae* and reported in other *Vibrio* strains.

Toxin	Microorganism	Main pathological effects	Main cellular effects	Reference
Cholera toxin	*V. cholerae*	Diarrhea	Activation of adenylate cyclase, increase in cAMP, active secretion of electrolytes, and water	Waldor and Mekalanos, 1996
	*V. mimucus*	Diarrhea?	Unkown	Shi et al., 1998
*Zot*	*V. cholerae*	*Diarrhea*	*Increase permeability of epithelial barrier by opening of tight junctions*	Fasano et al., 1991
	*V. parahaemolyticus*	Diarrhea?	Unkown	Castillo et al., 2018
	*V. vulnificus*	Diarrhea?	Unkown	Park et al., 2011
	*V. mimucus*	Diarrhea?	Unkown	Shi et al., 1998
Ace	*V. cholerae*	Diarrhea	Alters ion transport increasing electrolyte and water secretion	Trucksis et al., 1993
	*V. parahaemolyticus*	Diarrhea?	Unkown	Castillo et al., 2018
	*V. vulnificus*	Diarrhea?	Unkown	Park et al., 2011
	*V. mimucus*	Diarrhea?	Unkown	Shi et al., 1998
Heat stable enterotoxin (STa)	*V. cholerae*	Diarrhea	Increase in electrolyte and water secretion	Al-Majali et al., 2000
RTX	*V. cholerae*	Diarrhea	Pore formation Depolymerization of the actin cytoskeleton	Chatterjee et al., 2008
	*V. parahaemolyticus*	Diarrhea?	Unkown	Castillo et al., 2018
	*V. vulnificus*	Diarrhea	Pore formation Depolymerization of the actin cytoskeleton	Lee et al., 2007
Cholix toxin	*V. cholerae*	Extraintestinal infection	Inhibition of protein synthesis	Jørgensen et al., 2008
Hemagglutinin (HA)/protease (HA/P)^∗^	*V. cholerae*	Diarrhea	Mucinase, covalent modification of other toxins, and perturbs the paracellular barrier	Silva et al., 2006; Lutfullah et al., 2008


*^∗^Not a toxin but it has been described that it may play a role during colonization of the intestine.*

## Author Contributions

DP-R and KG conceived the idea. DP-R, KG and PN wrote the manuscript. LP and VJ made the figures. All authors read, discussed and approved the final version.

## Conflict of Interest Statement

The authors declare that the research was conducted in the absence of any commercial or financial relationships that could be construed as a potential conflict of interest.
